# Sulfated and Sulfur-Containing Steroids and Their Pharmacological Profile

**DOI:** 10.3390/md19050240

**Published:** 2021-04-24

**Authors:** Tatyana A. Pounina, Tatyana A. Gloriozova, Nick Savidov, Valery M. Dembitsky

**Affiliations:** 1 Far Eastern Geological Institute, Russian Academy of Sciences, 159 Prospect 100-letiya Vladivostoka, 690022 Vladivostok, Russia; pounta@mail.ru; 2 Institute of Biomedical Chemistry, 10 Building 8, Pogodinskaya Street, 119121 Moscow, Russia; tatyana.gloriozova@ibmc.msk.ru; 3 Centre for Applied Research, Innovation and Entrepreneurship, Lethbridge College, 3000 College Drive South, Lethbridge, AB T1K 1L6, Canada; nick.savidov@lethbridgecollege.ca; 4 A.V. Zhirmunsky National Scientific Center of Marine Biology, 17 Palchevsky Str., 690041 Vladivostok, Russia

**Keywords:** sulfated, sulfur-containing, epithio, steroids, pharmacology, antitumor, marine, PASS

## Abstract

The review focuses on sulfated steroids that have been isolated from seaweeds, marine sponges, soft corals, ascidians, starfish, and other marine invertebrates. Sulfur-containing steroids and triterpenoids are sourced from sedentary marine coelenterates, plants, marine sediments, crude oil, and other geological deposits. The review presents the pharmacological profile of sulfated steroids, sulfur-containing steroids, and triterpenoids, which is based on data obtained using the PASS program. In addition, several semi-synthetic and synthetic epithio steroids, which represent a rare group of bioactive lipids that have not yet been found in nature, but possess a high level of antitumor activity, were included in this review for the comparative pharmacological characterization of this class of compounds. About 140 steroids and triterpenoids are presented in this review, which demonstrate a wide range of biological activities. Therefore, out of 71 sulfated steroids, thirteen show strong antitumor activity with a confidence level of more than 90%, out of 50 sulfur-containing steroids, only four show strong antitumor activity with a confidence level of more than 93%, and out of eighteen epithio steroids, thirteen steroids show strong antitumor activity with a confidence level of 91% to 97.4%.

## 1. Introduction

Sulfur is one of the most abundant chemical elements in nature [1,2]. In addition, sulfur is found in many classes of organic compounds such as peptides, various terpenoids and steroids, and other compounds [1,2,3,4,5,6,7,8,9,10,11,12,13,14,15,16]. More than 3000 different sulfur compounds are found in various ecological niches, including oceans and freshwater lakes, volcanic gases, sediments, and rocks [12,13,14,15,16,17,18,19,20,21,22]. Many photosynthetic organisms such as algae and phytoplankton assimilate sulfur as sulfate. Sulfate is generally readily available in aquatic ecosystems for many marine and freshwater organisms [1,2,3,4,9,10,14,18,20,23,24,25,26,27]. The biochemistry and geochemistry of sulfur are currently well studied, and many publications are devoted to this topic [1,2,3,4,5,6,7,8,9,10,11,12,13,14,15,16,17,18,21,22,23,24,25,26,27,28,29,30,31,32,33,34,35,36,37,38,39,40,41,42].

Sulfated compounds, including steroids, are widely distributed in nature [1,2,3,4,5,6,7,8]. Sulfated steroids and triterpenoids are found in many animals, reptiles, and humans [9,10,11,12,13,14]. In addition, these compounds are present in extracts of plants and some shrubs, produced by microorganisms, fungi, and found in many marine invertebrates such as brittle star, starfish, sponges, ascidian, snails, and algae [2,15,16,17,18,19,20,21].

Studies of the last decades of marine sediments have shown that extracts from these geological formations contain a wide variety of steroids, terpenoids, and other lipids [22,23,24,25,26,27,28,29]. These findings indicate that such a variety of lipids is of organic origin, and, apparently, the producers are bacteria, terrestrial higher plants, unicellular and diatom algae, marine invertebrates, and other organisms [22,23,30]. Interestingly, sterols, triterpenoids, and even boron-containing compounds were found in marine sediments of the Cretaceous period more than 150 million years ago [31,32,33,34,35,36,37,38]. Sulfur-containing steroids and terpenoids are found in ancient earth rocks, crude oil, and other marine and aquatic sediments [39,40,41,42]. There is still no convincing evidence that microorganisms are not involved in the formation of sulfur-containing steroids and terpenoids, but, nevertheless, these lipid markers are of great interest for geochemistry, biochemistry, medicinal chemistry, and pharmacology [43,44,45].

This review focuses on sulfated steroids and triterpenoids that have been found and isolated from marine sources such as algae, sponges, and other marine invertebrates. The review also presents sulfur-containing steroids and triterpenoids isolated from soft corals, marine sediments, and crude oil and other geological deposits. In addition, we have included in this review several semi-synthetic and synthetic epithio steroids, which represent a rare group of bioactive lipids and have not yet been found in nature but exhibit a high level of antitumor activity. For all natural, semi-synthetic and synthetic compounds included in this review, their pharmacological profile and specific diversity of biological activity are presented.

## 2. Mono Sulfated Steroids Derived from Marine Sources

It is known that sulfated steroids are of potential interest for practical and clinical medicine, due to the fact that many of them exhibit a wide range of biological activities; however, the activity of many sulfated steroids has not been determined [2,9,10,15,16,17,18,21,46].

**Figure 1:**
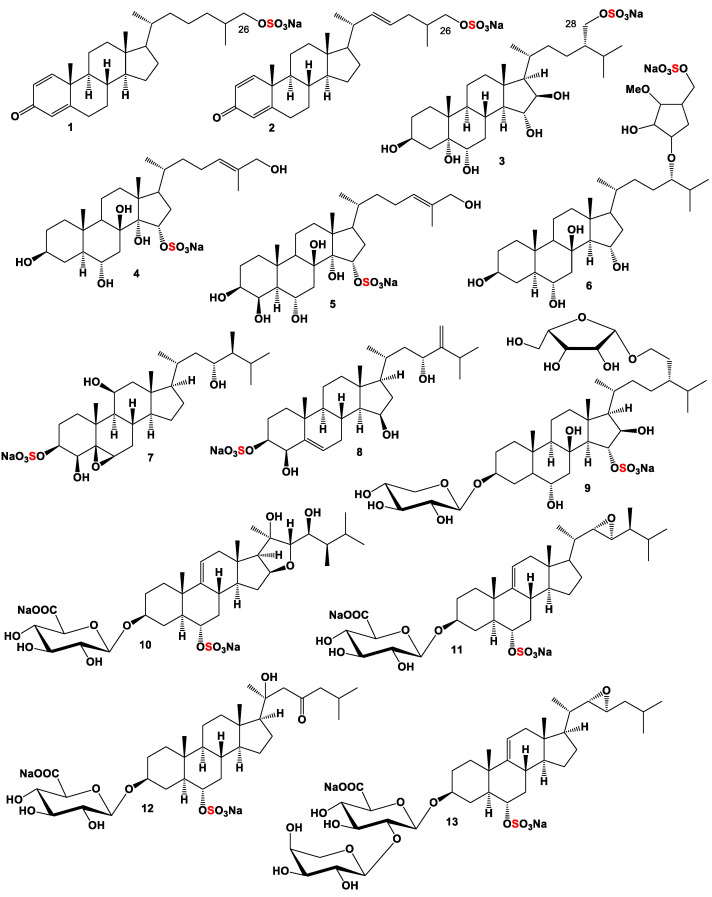
Mono sulfated steroids are derived from marine sources, and their pharmacological profile is shown in Table 1.

Two sulfated steroids, (**1**, for for structure see Figure 1 and for activities see Table 1) and (**2**), were obtained from a lipophilic extract of an undescribed bryozoan species in the genus *Calyptotheca*. Both isolated metabolites exhibited moderate cytotoxicity at IC_50_ 54 and 30 µM, respectively, against the rat bladder carcinoma epithelial NBT-T2 cell line [47].

Several sulfated steroids have been found and isolated from the marine starfish. Thus, sulfated polyhydroxysteroid named microdiscusol G (**3**) was isolated from the Arctic starfish *Asterias microdiscus*. The 28-sulfooxy-24-methylcholestane side chain in (**3**) has been found among starfish steroid metabolites for the first time [48]. Two steroids (**4** and **5**) and an unusual glucoside (**6**) were isolated from the starfish *Archaster typicus* collected in shallow waters of the Quang Ninh province (Vietnam). Isolated compounds showed moderate toxic effects in the sperm and 8-blastomere tests on embryonal development of the sea urchin *Strongylocentrotus intermedius* [49,50].

Polyoxygenated steroids belonging to a new structural group of sponge steroids called gracilosulfates B (**7**) and D (**8**) were isolated from the marine sponge *Haliclona gracilis*. Both compounds inhibited the expression of prostate-specific antigen in a human prostate carcinoma epithelial tumor cell lines (22Rv1) [51]. Steroid glycoside, granulatosides D (**9**), belonging to the group of bi- and monoglycosides of polyhydroxysteroids, respectively, was isolated from the ethanolic extract of the starfish *Choriaster granulatus*. The obtained compound showed immunomodulatory properties, increasing the intracellular ROS (reactive oxygen species) level in peritoneal murine macrophages by 20% and decreasing intracellular ROS level by 21% in pre-treated with endotoxic lipopolysaccharide from *E. coli* peritoneal macrophages [52].

Several starfish polar steroids, downeyosides A (**10**), C (**11**), J (**12**), and H (**13**) sulfated steroid glucuronides, were isolated from the starfish *Henricia downeyae* (family Echinasteridae) collected in the northern Gulf of Mexico [53,54].

**Figure 2:**
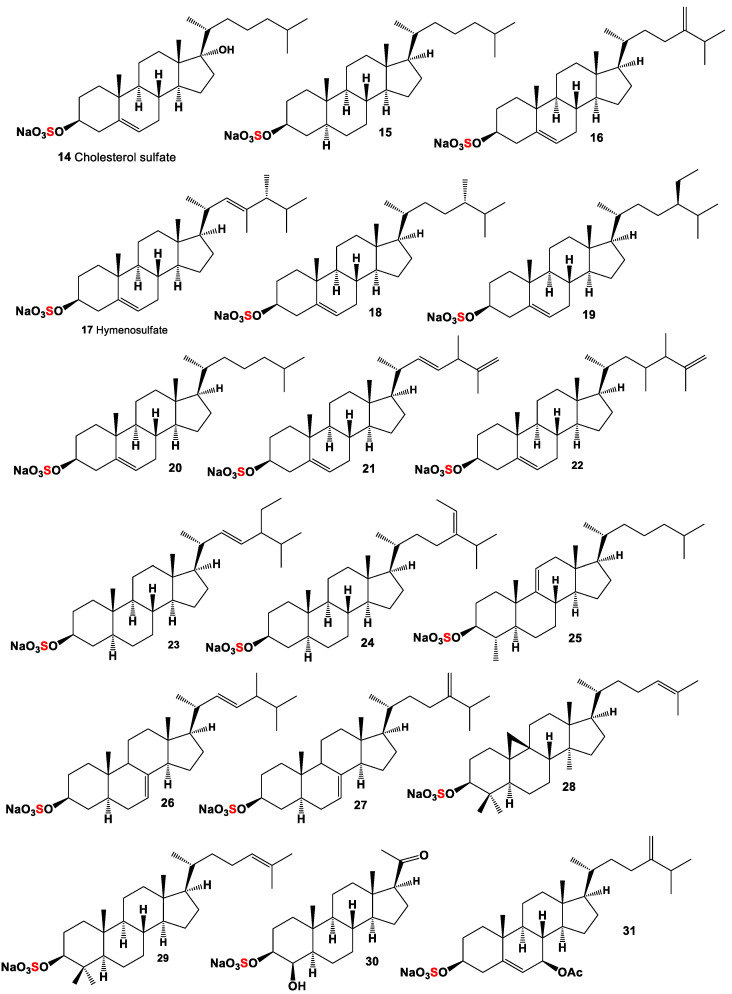
Mono sulfated steroids are derived from marine sources, and their pharmacological profile is shown in Table 2.

Cholesterol sulphate (**14**, structure see Figure 2 and activities see Table 2) has been isolated from the starfish *Asterias rubens* [55,56], and compound (**14**) and cholestanol sulphate (**15**) were identified as the major components among the 34 different sterol sulfates of the sea cucumber *Eupentacta fraudatrix* [57].

The 24-methylidene-cholesterol sulfate (**16**) was detected in the diatomic microalga *Nitzchia alba* more than 50 years ago [58], and another derivative of cholesterol sulfate called hymenosulfate (**17**) with an unusual side chain was found in the haptophytic microalga *Hymenomonas* sp. [59]. Three sulfated sterols, (**14**), 24-methylcholest-5-en-3β ol sulfate (**18**), and 5-sitosten-3β-ol sulphate (**19**) were produced by the diatom *Skeletonema marinoi* [60]. The cholest-5-ene-3β-sulfate sodium (**20**) was found in the methanol extract of the sea urchin *Diadema savignyi* [61].

Two derivatives of cholesterol sulfate (**21** and **22**) were isolated from the tropical marine cucumber *Holothuria* sp. [62], and two derivatives of cholestanol sulfates (**23** and **24**) and minor sterols (**25**–**29**) were detected in the Far Eastern holothurian *Eupentacta fraudatrix* [57].

**Figure 3:**
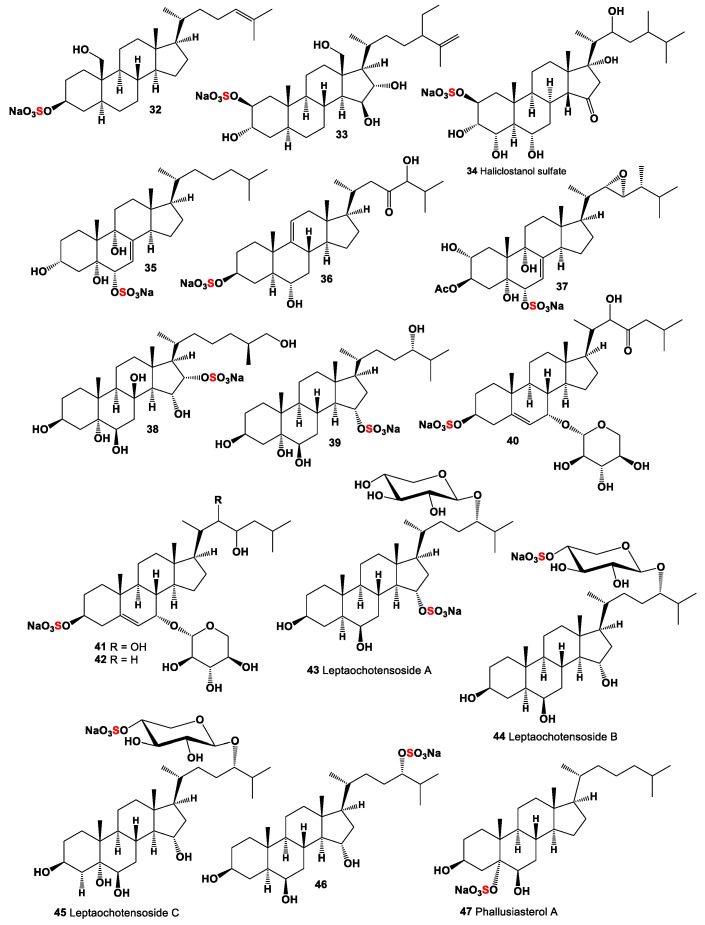
Mono sulfated steroids are derived from marine sources, and their pharmacological profile is shown in Table 3.

An Australian marine sponge *Stilopus australis* produced a sulfated steroid with the pregnane skeleton (**30**) [63], and annasterol sulfate (**31**) was isolated from the Pacific deep-water sponge *Poecilastra laminaris*, and this compound showed a β-1,3-glucanase inhibitor [64]. Polyhydroxy-steroid monosulphate (**32**, for structure see Figure 3 and for for activities see Table 3) was found in the extract of the sponge *Toxadocia zumi* [65]. Rare mono sulfate steroid with the sulfate group in 2β-position (**33**) was isolated from the sponge *Echinoclathria suhispida* collected from the Japan Sea near the coast of Japan [66]. The Malaysian sponge *Haliclona* sp. from an Indo-Pacific has yielded haliclostanone sulphate (**34**) [67]. Cytotoxic steroid (**35**) containing a sulfate group in 6α-position was found in the sponge *Dysidea fragilis* collected from the lagoon of Venice, Italy [68]. A sulfated steroid called apheloketotriol (**36**) was isolated from a Far Eastern sponge *Aphelasterias japonica* [69], and acanthosterol E (**37**) was found in the sponge *Acanthodendrilla* sp. containing sulfate group in 6-position [70].

Rare mono sulfate (**38**) containing a sulfate group in 16-position was found in starfish *Luidia clathrata* (family Luidiidae) [71], although the other 3-monosulfate (**39**) was isolated from Far Eastern starfish *Luidia quinaria* (Japan Sea) [72]. Three sulfated steroidal glycosides (**40**–**42**) were isolated from the visceral extract of the cone snail *Conus pulicarius*. The three new compounds exhibited significant in vitro cytotoxicity (GI_50_ values down to 0.49 µM) against the K562 human leukemia cell line [73]. Three sulfated steroid monoglycosides, leptaochotensosides A–C (**43**–**45**), and a sulfated polyhydroxylated steroid (**33**) were isolated from the alcoholic extract of the Far Eastern starfish *Leptasterias ochotensis* [74]. Leptaochotensoside A (**43**) demonstrated inhibition of T-47D cell colony formation in a soft agar clonogenic assay at nontoxic doses. In addition, this compound decreased the epidermal growth factor induced colony formation of mouse epidermal JB6-Cl41 cells. The cancer preventive action of (**43**) is realized through regulation of the mitogen-activated protein kinase signaling pathway.

**Figure 4:**
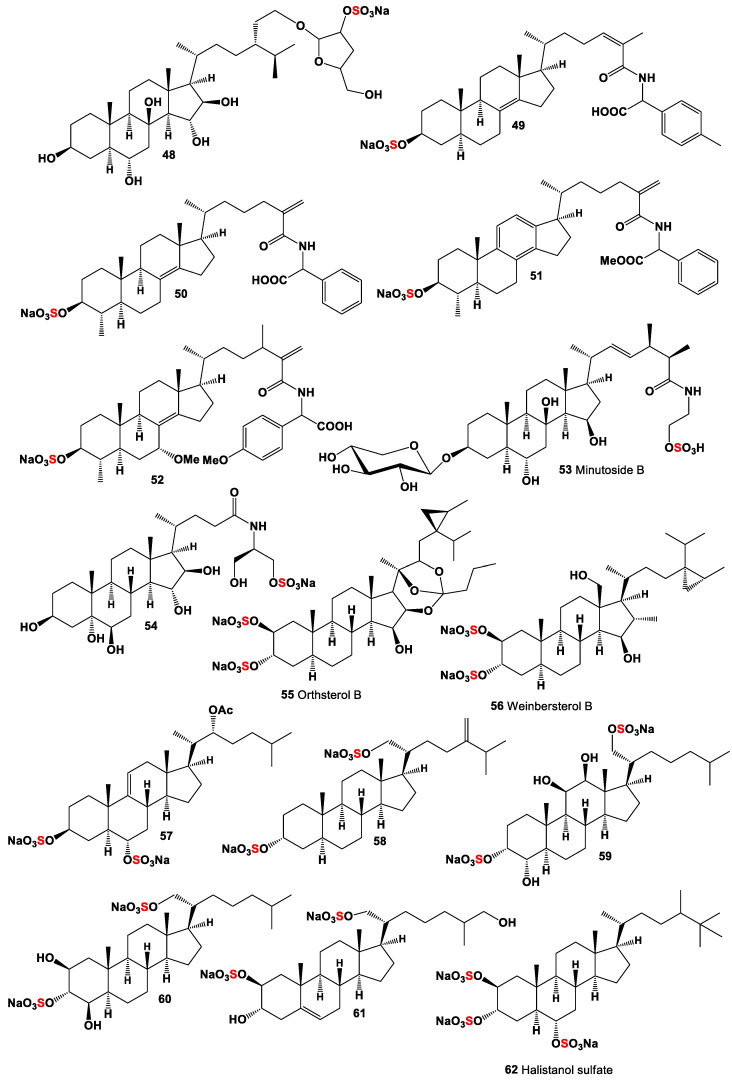
Mono- and poly-sulfated steroids derived from marine sources, and their pharmacological profile, are shown in Table 4.

A rare sulfated steroid at position 5, named phallusiasterol A (**47**), was isolated from the Mediterranean ascidian *Phallusia fumigate* [75]. A polyhydroxylated sterol called asterosaponin P2 (**48**, for structure see Figure 4 and for activities see Table 4), with the sulfate group only in the side chain, isolated from the Far-Eastern starfish *Patiria* (*Asterina*) *pectinifera* [76], exhibited activity against HSV-1 [77].

A series of sulfated steroid-containing amide fragments (**49**–**54**) was detected in marine sponges and echinoderms. Thus, starfish, *Styracaster caroli*, which was collected at a depth of 2000 m between the islands of Thio and Lifou (New Caledonia), contained unique polyhydroxylated steroids in water–acetone extract (**49** and **50**) [78]. The same steroids (**49**–**52** and **54**) were found in the sponge *Polymastia boletiformis* [79,80]. Sulfated steroid xyloside, minutoside B (**53**), has been isolated from the ethanolic extract of the starfish *Anasterias minuta*. This xyloside exhibited antifungal activity against *Cladosporium cucumerinum* and *Aspergillus flavus* [81].

## 3. Di- and Poly-Sulfated Steroids Derived from Marine Sources

Natural di- and poly-sulfates of steroids represent a rare group of bioactive lipids. Their total content in marine organisms is two to three times less than that of steroids containing one sulfate group [2,16,18,43]. The antiviral orthoesterol B (**55**) showed antiviral activity was found in the marine sponge *Petrosia weinbergi* [82]. Weinbersterol B (**56**), a sulphated tetrahydroxy steroid with an unprecedented cyclopropane-containing side chain, was isolated from the sponge *Petrosia weinbergi*. This compound is active in vitro against feline leukemia virus and active in vitro against HIV [83].

Several steroid disulfates were found in starfish and ophiuroids. Thus, compound (**57**) was isolated from the starfish *Tremaster novaecaledonia* [84] and from *Aphelasterias japonica* [85]. Brittle stars that are echinoderms and belong to the class Ophiuroidea produce many active metabolites including plasmalogen lipids, fatty acids, and steroids [86,87,88,89,90,91]. Sulfated stanols are widely distributed in various representatives in more than 30 species of Ophiuroidea [2,15,16,17]. Disulphate stanol (**58**) was first discovered in brittle star *Ophioderma longicaudum* [92], and another steroid (**59**) containing an additional hydroxy group at C12 was isolated from *O. longicaudum* from the Mediterranean Sea [2,16,17,93]. The disulfated steroid (**60**) containing three functional groups in the ring A was isolated from the Antarctic brittle star *Ophiosparte gigas* [17], and the same steroid also was found in *Astroclades exiguus* and *Amphiophiura ponderosa* [94]. Rare sulfated steroid (**62**), containing sulfate groups in 2β- and 21α-positions, was detected in extracts from the starfish of the starfish *Pteraster tesselatus* [95].

**Figure 5:**
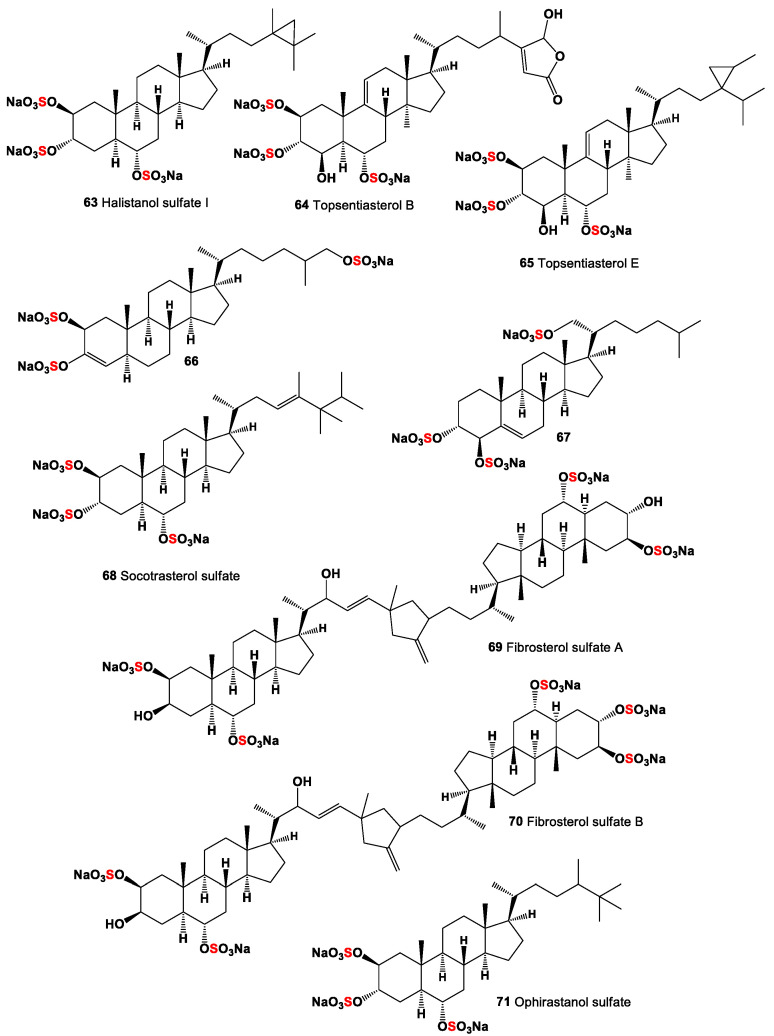
Polysulfated steroids derived from marine sources, and their pharmacological profile is shown in Table 4.

Trisulfated polyhydroxysteroids are rare and typical metabolites that are produced by marine sponges and echinoderms [2,16,18,19,93]. Halistanol sulphate sodium (**63**, for structure see Figure 5 and for activities see Table 4), the most widespread sponge steroid sulfate, was discovered from the sponge *Halichondria moori* by Fusetani and co-workers [96]. Analog of compound (**63**), halistanol sulfate I sodium (**64**) was isolated from a marine sponge *Halichondria* sp. collected at Hachijo-jima Island. The obtained steroid showed inhibitory activity against SIRT 1-3 with IC_50_ of 45.9, 18.9, and 21.8 μM, respectively [97].

A polyhydroxylated sterol derivative called topsensterol B (**65**) has been isolated from a marine sponge *Topsentia* sp. collected from the South China Sea. The isolated compound exhibited cytotoxicity against human gastric carcinoma cell line SGC-7901 with an IC_50_ value of 8.0 μM [98], and topsentiasterol sulphate E (**66**) was found in extracts of the sponge *Spheciospongia* sp., collected in the Philippines. This compound inhibited PKCzeta with an IC_50_ value of 1.21 µM, and in a cell-based assay also inhibited NF-kappa B activation with EC_50_ value of 12 µM [99].

Representatives of Echinodermata’s and other marine and freshwater invertebrates contain very interesting lipid molecules, such as polar lipids and fatty aldehydes [86,100,101,102,103,104]. In addition, two trisulfated steroids have also been found in various types of ophiuroids, so the steroid (**67**) is isolated from *Ophiura sarsi*, and another steroid (**68**) is isolated from *Ophiorachna incrassate* [105].

Rare sulfated sterol dimers called fibrosterol sulphates A (**69**) and B (**70**) were isolated from a *Lissodendoryx* (*Acanthodoryx*) *fibrosa* sponge from Coron Island Palawan, Philippines [99]. Both compounds have inhibited PKC ζ with IC_50_ values of 16.4 and 5.6 μM, respectively [106]. Socotrasterol sulphate (**71**) was isolated from different sponge species [107], and ophirapsterol sulphate (**69**) was found in *Topsentia ophiraphidites* [108].

## 4. Sulfur-Containing Steroids Derived from Marine and Terrestrial Sources

A wide variety of chemical structures of steroids and terpenoids was found in extracts of marine sediments, as well as both contemporary and geologically ancient deposits [29,31,109,110,111,112,113,114]. Aromatic sterols, triterpenoids, and sulfur-containing compounds have been found in marine sediments and crude oil [31,32,33,34,115,116,117,118,119,120].

Until now, there has been no evidence that microorganisms do not participate in the formation of sulfur-containing steroids, but nevertheless, these lipid markers are of great interest for biochemistry, geochemistry, and pharmacology [119,120,121,122]. More than 3000 terpenoids and other lipid molecules have been isolated from sedimentary rocks and marine sediments—this is an established fact [28,30,33,39,40,41,42,111,112,113,114]. Sulfur-containing steroids and terpenoids make up the bulk of natural compounds that are isolated from ancient sediments, petroleum, deposits, and marine sediments [111,112,113,114,117,123,124,125,126,127].

**Figure 6:**
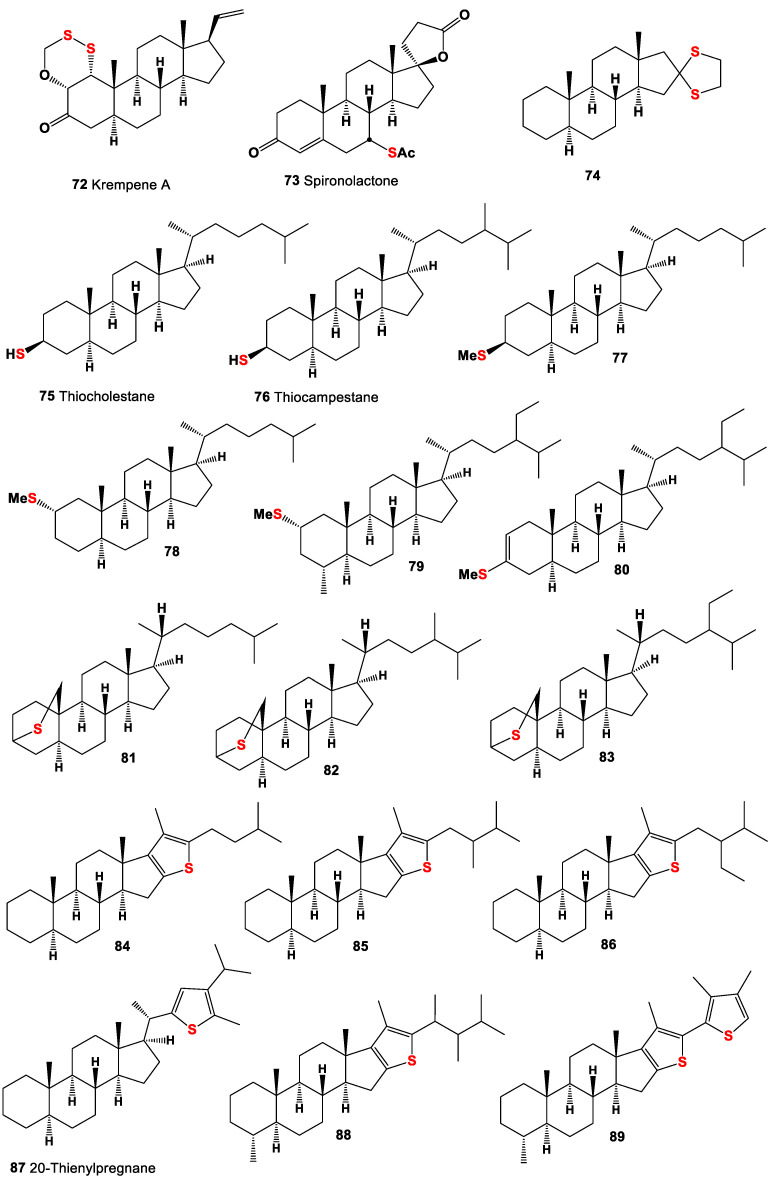
Sulfur-containing steroids and triterpenoids derived from marine invertebrates, sediments, crude oil, and other sources, and their pharmacological profile is shown in Table 5.

An unusual pregnane-type steroid called krempene A (**72**, structures are shown in Figure 6 and activity showed in Table 5) was isolated from the marine soft coral *Cladiella krempfi*. The isolated compound contains a very unusual structural motif, with a hexacyclic oxadithiino unit fused to the steroidal ring A [128].

The 3-(3-oxo-7α-methylsulfinyl-6β,17β-dihydroxy-4-androsten-17α-y1)-propionic acid γ-lactone called spironolactone (**73**) has been found and isolated in the human urine by Karim and Brown in 1972 [129]. The physiological properties and biogenesis of spironolactone in humans are well described in several reviews [130,131,132,133].

5α-Androstan-16-one, cyclic ethylene mercaptole (**74**), was present in the ethanol extract of the whole plant of *Andrographis echioides* [134]. Two thiosteranes, 3β-thio-5α-cholestane (**75**), and 3β-24-methyl-5α-cholestane (**76**) were isolated from extracts from Marrakech activated sludge, which is formed from the leaves and the date palm of the date palm [135,136].

Four thio-5α-cholestane derivatives (**77**–**80**) were isolated from the polar fractions of the organic matter of both oils and sediment extracts, which were collected in various parts of the world, in Europe, North America, and China [113,114,137,138,139,140].

**Figure 7:**
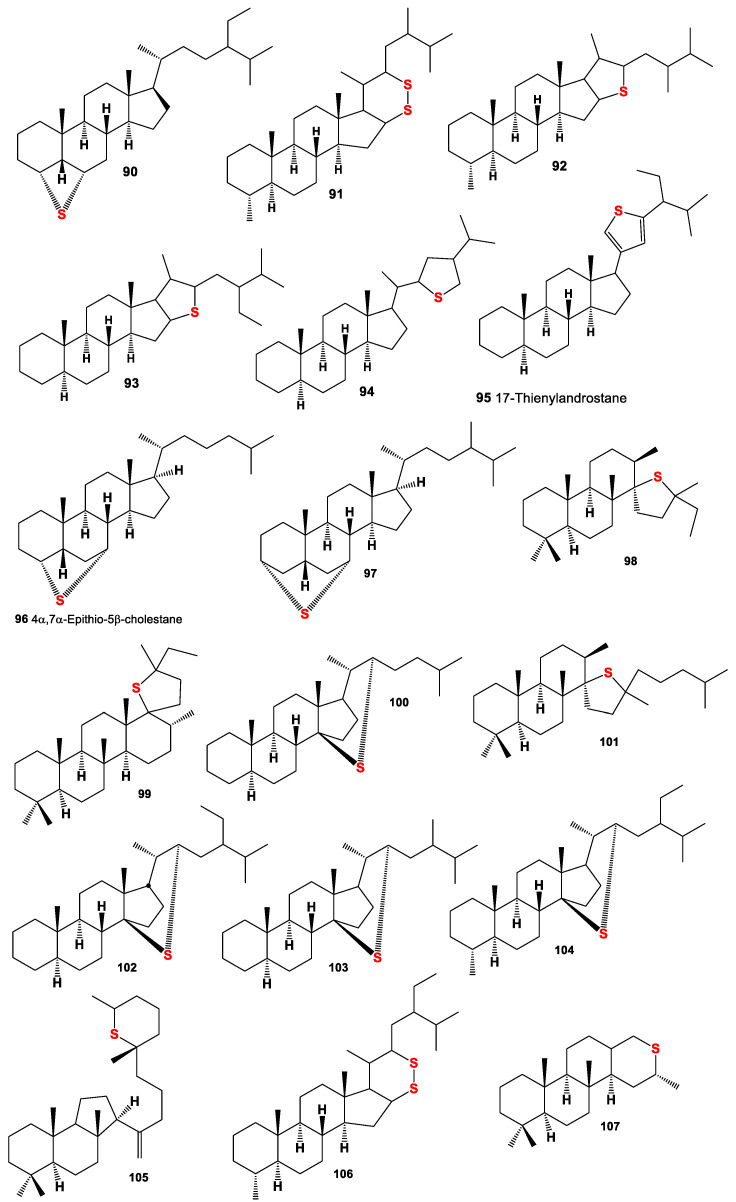
Sulfur-containing steroids and triterpenoids derived from marine sediments, crude oil, and other sources, and their pharmacological profile is shown in Table 6.

Many authors have attempted to investigate the organic matter generated in hypersaline environments, including both sea and salt seas, and a warm eutrophic salt lake in Southern California, where sulfur-containing compounds have been found and isolated, including unusual thiosterols (**81**–**87**), and their unusual chemical structures have been established [113,114,124,125,126,127]. Interesting data were obtained by Chinese scientists who studied the sulfur-rich heavy oils in Jinxian Sag, Bohai Bay Basin, northern China, and found high abundances of organic sulfur compounds, including a series of short-chain steranes (C21-26), unusual short chainlanostanes (C24-25), 4-methyl steranes (C22-23), 4,4-dimethyl steranes (C22-24), and androstanes (C19-20), accompanied by high-molecular-weight analogues, regular steranes, 4-methyl steranes, and 4,4-dimethyl steranes, as well as sulfur containing steroids (**80**,**88**–**92**, for structure see Figure 6 and Figure 7 and for activities see Table 5 and Table 6). The authors believe that the occurrence of abundant sulfur-containing steroids was the result of extensive sulfurization during early diagenetic stages, because many more double bonds, hydroxyl groups, and carbonyl groups exist in sterols and steranes, which are prone to attack by inorganic sulfur [141].

Several series of organic sulfur compounds have been identified in several oils and sediment extracts including Rozel Point Oil (Box Elder County, UT, USA, Miocene). Series of isoprenoid thiophenes, isoprenoid thiolanes, isoprenoid bithiophenes, isoprenoid thienylthiolanes, isoprenoid benzothiophenes, 2,5-di-n-alkylthiolanes, 2,6-di-n-alkylthianes and 2,4-di-n-alkyl-benzo(b)thiophenes, and several thiophene and thiolane steranes (**84**–**86**, **92**, **93** and **95**) have been identified [142]. A sulfur-containing sterane, 4α,7α-epithio-5β-cholestane (**96**), has been identified in a sediment extract from the Miocene Northern Apennines marl (Italy) [143]. Another 3α,7α-epithio-24-Me-5β-cholestane (**97**) was isolated from the polar fractions of sediment extracts and a crude oil taken from a Jurfed Darawish oil shale Jordan [144]. C27–C29 homologs have been detected in sediment extracts of three different formations and in one petroleum sample. These sulfur-containing steroids are probably formed by an intramolecular reaction of inorganic sulfides with early diagenetic products of Δ5,7-sterols.

Several sulfur-containing compounds (**86**, **92**, **94**, **97**, **99**, **102**, and **104**) were isolated from water–methanol extracts of the organic phase of solutions obtained from treatment with microbial mats, which were deposited in a Lagoona setting, revealing three lithofacie, and limestones with fine-scale parallel laminations, limestones with undulated (stromatolite-type) laminations, and massive limestones. These different lithofacies refer to The Upper Jurassic Calcaires en plaquettes Formation, which is located on the southern Jura in France [145]. The triterpenoid thiane (**105**) was identified from organic matter in the Holocene and latest Pleistocene sediments of the Cariaco Basin, Venezuela [146].

**Figure 8:**
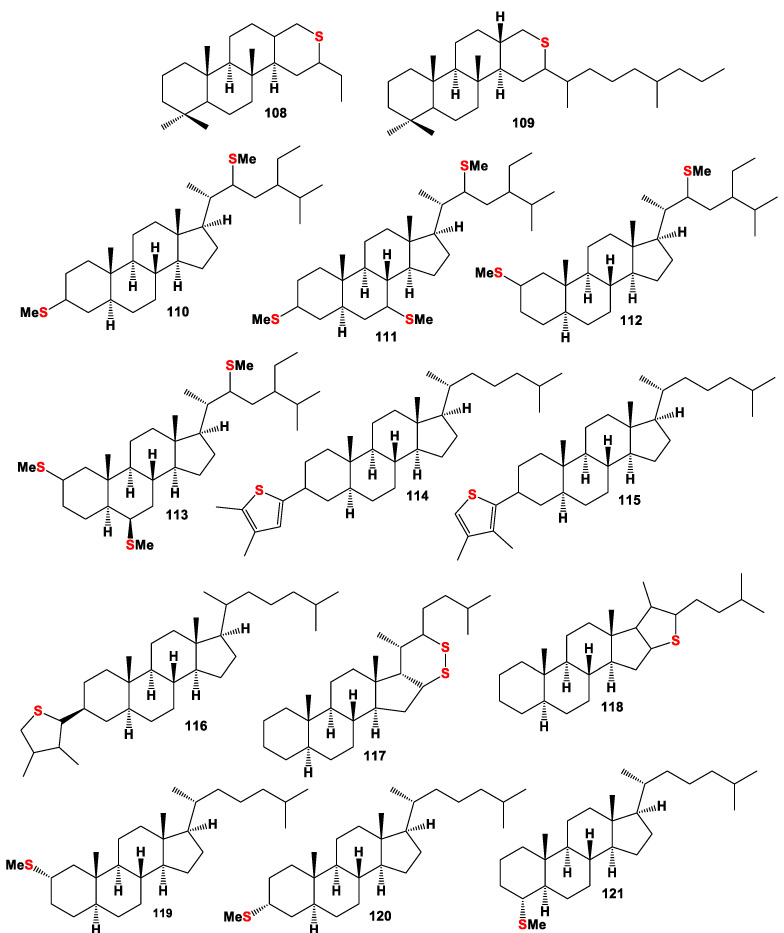
Sulfur-containing steroids and triterpenoids derived from marine sediments, crude oil, and other sources, and their pharmacological profile is shown in Table 7.

A series of sulfur-containing sterols (**98–101**, **103**, **107,** and **108**, for structure see Figure 8 and for activities see Table 7) have been characterized in a wide range of sediments, and they were isolated from a sample from Sémecourt (Paris basin, France) [147], and compound (**109**) was detected in petroleum from Alberta province, Western Canada [148].

Sulfur-containing steroids (**84**, **85**, **89**, **90**, **117,** and **118**) and methylthio-steroids (**110**–**113**) were identified by GC-MS in saturate hydrocarbon fractions of heavy oil with a high sulfur content in the Jinxian Sag, Bohai Bay Basin, North China [139,141]. Homologous series of 3-n-alkyl-1,2-dithianes and 3-n-alkyl-6-methyl-1,2-di-thianes, including (**91**, **106**, and **117**), have been identified in immature sediments [137].

A series of thiophenes with C-3 alkylated steroid carbon skeletons (**114**–**116**) have been identified in sediments of the Miocene Monterey Formation (California, USA) and in the Turonian Tarfaya basin (Morocco). Their carbon skeletons were unusual in the sense that the alkyl sidechains at C-3 are almost exclusively isopentyl, 3-methylpentyl, and 2,3-dimethylbutyl moieties, whilst n-alkyl (pentyl or hexyl) moieties are almost absent [144].

2α-, 3α-, and 4α-Methylthio-steroids (**119**–**121**) that were present in the polar fractions of six immature samples (both crude oils and sediment extracts) have been analyzed using S-selective chemolysis methods and analytical pyrolysis [137].

## 5. Epithio Steroids

Semi-synthetic and synthetic epithio steroids represent a rare group of bioactive lipids, since they are hydrophobic molecules insoluble in water, which were not found in nature. Epithio steroids have been reported to possess a variety of cytotoxic activities, and they are widely used as anticancer agents. The thiirane group is an important substance and shows some promising biological activities.

Steroids containing an epithio group in positions 2 and 3 belong to anabolic steroids and are widely known and used in sports medicine and are of great interest for the pharmacology of sports and other aspects of medicine [149,150,151,152,153,154]. The most widely known are such epithio steroids that are used in sports pharmacology and medicine: epistane (2α,3α-epithio-17α-methyl-5α-androstan-17β-ol), epitiostanol (2α,3α-epithio-5α-androstan-17β-ol, known as potent anti-estrogenic and antitumor agent), hemapolin (2α,3α-epithio-17α-methyl-5α-androstan-17β-ol), mepitiostane (epitiostanol 17β-methoxy-cyclopentyl ether), epivol (2α,3α-epithio-17α-methylethio-allo-cholanol), epivol black (2,3α-epithio-17α-methyl-5α-androstan-17β-ol), and straight epi (2,3α-epitio-17α-methyl-etioallocholane-17β-ol) [149,150,152,155,156,157,158].

**Figure 9:**
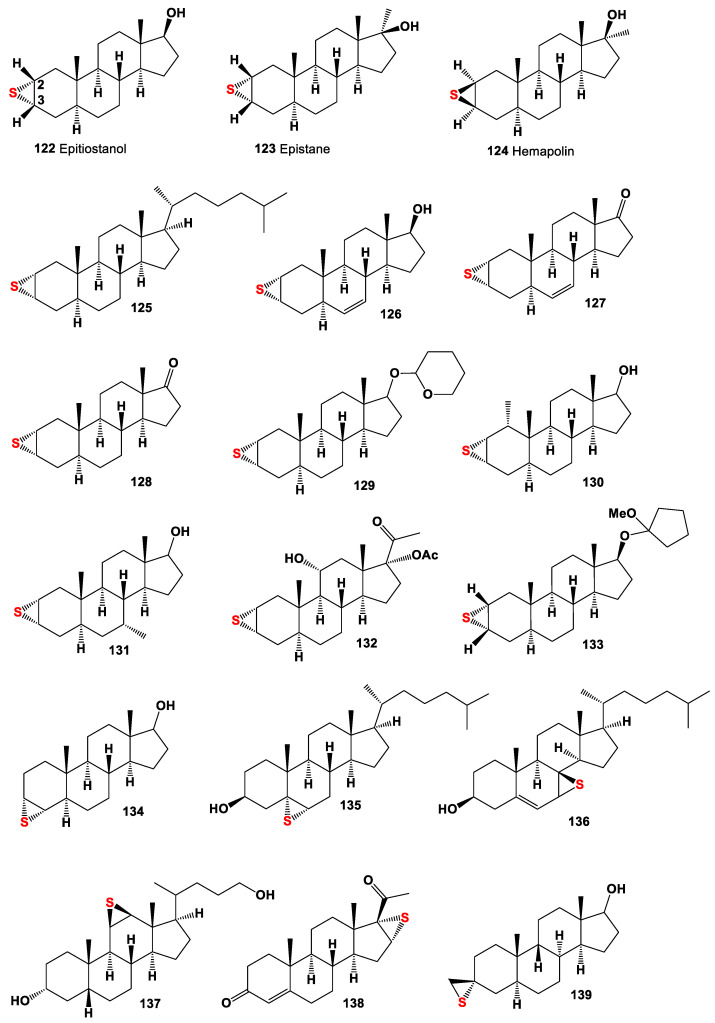
Semi-synthetic and synthetic epithio steroids, and their pharmacological profile is shown in Table 8.

2,3-Epithio steroids (**122**-**133**, for structure see Figure 9 and for activities see Table 8) belong to a large group of anabolic steroids and are of the greatest interest to pharmacologists and lipidomic networks. Two known 2,3-epithio steroids, such as epitiostanol and epistane methylated prohormone, were both synthesized in the 1960’s and used as a treatment for breast cancer, and the second steroid was used to increase lean muscle mass as well as cutting fat [159,160,161,162]. The 2,3-epithio steroids have exhibited other specific physiological activities [152]. Several epithio steroids such as two 3,4-epithio steroids, 3β,4β-epithio-5α-androstan-17β-ol (**134**), and 5α,6α-epithio cholestan-3β-ol (**135**), 7α,8β-epithio-3β-cholesterol (**136**), 11β,12β-epithio- (**137**), 17α,18α-epithio- (**138**) steroids, and an interesting thiirane-containing steroid (**139**) have been synthesized in different laboratories; however, they had one goal of obtaining a steroid with specific biological activities, for example, an inhibitor of estrogen synthetase, gonadotropin inhibitors, or agents that demonstrate antiseptic, germicidal, fungicidal, or antitumor activities [152,160,162,163,164].

## 6. Comparison of Biological Activities of Sulfated and Sulfur-Containing Steroids

The concept, which was formed more than 150 years ago, that the biological activity of natural and synthetic compounds depends on their chemical structure has been confirmed at the current time [165]. Using this concept, it is generally accepted that the biological activity of both natural and synthetic compounds depends on their chemical structure [166,167]. Apart from the sharp jumps in biological activity that are observed for some medicinal compounds [168], this can be considered a violation of this rule; however, for most chemical compounds, the structure–activity ratio (SAR) is widely used in medicinal chemistry and pharmacology to search for and optimize new pharmacological agents [169].

Software PASS is the first software for in silico estimation of biological activity profiles [170], of which the development was started more than 30 years ago [171]. Its current implementation predicts about 10,000 pharmacological effects, molecular mechanisms of action, pharmacological effects, toxicity, side effects, anti-targets, transporters-related interactions, gene expression regulation, and metabolic terms [166]. Due to the utilization of chemical descriptors that reflect the essential features of ligand–target interactions and a robust mathematical approach for analysis of structure–activity relationships, the average accuracy of PASS predictions was 96% [172]. Based on the PASS predictions provided by the appropriate web-service [173], over 29,000 researchers from 105 countries selected the most promising virtually designed molecules for synthesis and determined the optimal directions for testing their biological activity [174,175,176,177].

In this study, PASS predictions were used to estimate the general pharmacological potential for the analyzed natural, semi-synthetic, and synthetic sulfated and sulfur-containing steroids, and triterpenoids. For about ten thousand pharmacological effects and molecular mechanisms of action, probabilities of belonging to the class of “active” Pa, varied from zero to one, were estimated. PASS estimates are presented as Pa values, which correspond to the probability of belonging to a class of “actives” for each predicted biological activity. The higher the Pa value is, the higher the probability of confirming the predicted activity in the experiment. On the other hand, estimated Pa values might be relatively small for some activities if the analyzed molecule is not like the active compounds from the PASS training set. Pa values are indicated for all steroids and triterpenoids presented in this article in Table 1, Table 2, Table 3, Table 4, Table 5, Table 6, Table 7 and Table 8.

Since the end of March 2021, a new version of the PASS program has been used, with an increased amount of both natural and synthetic compounds, and, accordingly, the database of biological activities has increased.

### 6.1. Antitumor Activity of Natural Mono-, Di-, and Poly-Sulfated Steroids

**Figure 10:**
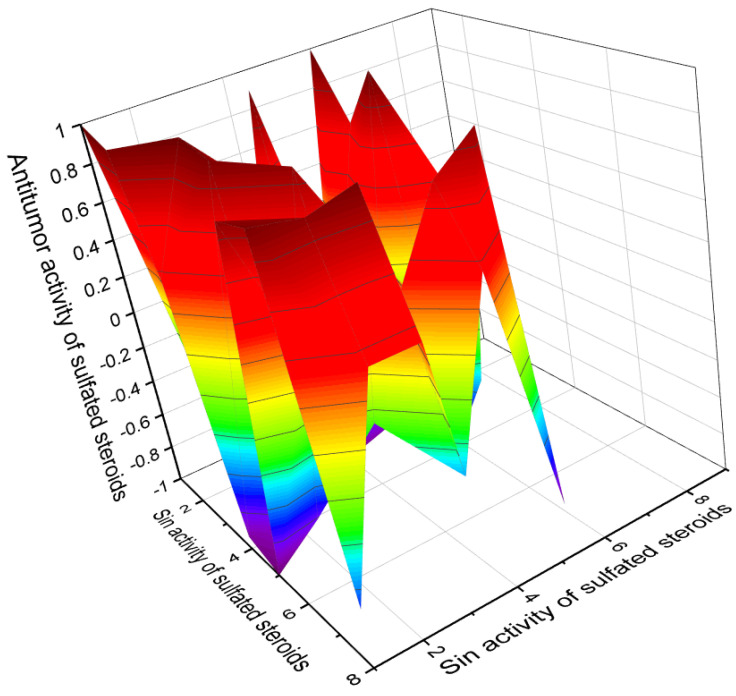
The 3D graph shows the predicted and calculated antitumor activity of selected sulfated steroids (compound numbers: **28**, **29**, **40**, **42**, **43**, **44**, **45**, **48**, and **64**) showing the highest degree of confidence, more than 91%. These sulfated steroids derived from marine sources can be used in clinical medicine as agents with strong antitumor activity.

**Figure 11:**
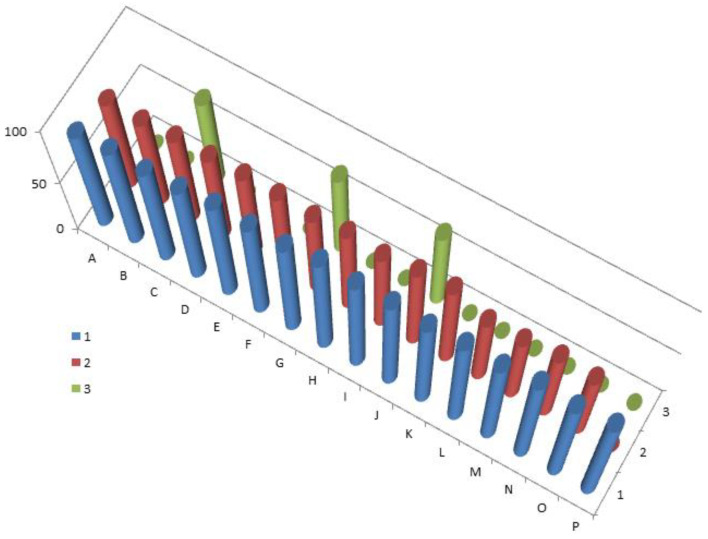
3D column graph of sulfated steroids derived from marine sources that show anti-hypercholesterolemic activity. The letters represent the steroid numbers shown in Figure 1, Figure 2, Figure 3 and Figure 4 and Table 1, Table 2, Table 3, Table 4 and Table 5: A—(**1**), B—(**14**), C—(**16**), D—(**18**), E—(**19**), F—(**20**), G—(**21**), H—(**23**), I—(**24**), J—(**26**), K—(**31**), L—(**40**), M—(**41**), N—(**42**), O—(**43**), and P—(**52**). Steroids that belong to this group, according to the data obtained by the PASS, have confirmed more than 90% of their biological activity.

**Figure 12:**
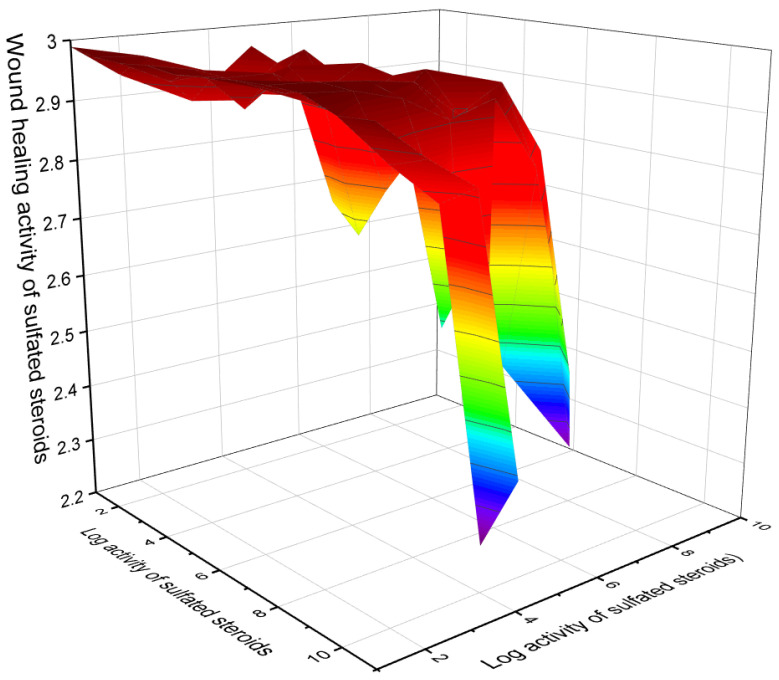
The 3D graph shows the predicted and calculated wound-healing activity of selected sulfated steroids (compound numbers: **14** (95.5%), **18** (93.8%), **19** (95.2%), **21** (92.8%), **29** (96.5%), **40** (95.3%), **41** (96.3%), **42** (97.5%), **43** (98.0%), **44** (97.7%), **60** (93.3%), and **61** (94.2%). These sulfated steroids show the highest degree of confidence, more than 92%.

Currently, about 7000 articles have been published covering various aspects of marine and terrestrial sulfated steroids and triterpenoids. Analyzing the data obtained using PASS compounds presented in this review, it can be stated that out of 71 marine sulfated steroids and triterpenoids, the activity is estimated with Pa from 68.6% to 94.8%, and only thirteen marine sulfated steroids demonstrate strong antitumor activity with a reliability of 90% to 94.8% (see Table 1, Table 2, Table 3 and Table 4 and structures shown in Figure 1, Figure 2, Figure 3, Figure 4 and Figure 5). However, most sulfated steroids exhibited moderate antitumor activity with 68% to 90% confidence. A 3D graph of the predicted antitumor and related activities is shown in Figure 10. In addition, many sulfated steroids exhibit moderate anti-hypercholesterolemic activity, and some steroids exhibit strong anti-hypercholesterolemic activity with a confidence level greater than 90% (see Figure 11). An interesting finding was that some sulfated steroids show wound-healing properties with more than 90% confidence, and this data is presented in Figure 12. Several sulfated steroids such as **1** (93.1%), **2** (92.7%), and **21** (92.8%) exhibit hemostatic properties with more than 92% confidence, and other steroids 29 (93.3%), 32 (93.3%), **34** (96.8%), **48** (92.4%), **65** (91.5%), **68** (91.5%), **69** (91.8%), **70** (93.4%), and **71** (93.4%) show hepatoprotective properties (see Table 1, Table 2, Table 3 and Table 4).

### 6.2. Biological Activity of Sulfur-Containing and Epithio Steroids

**Figure 13:**
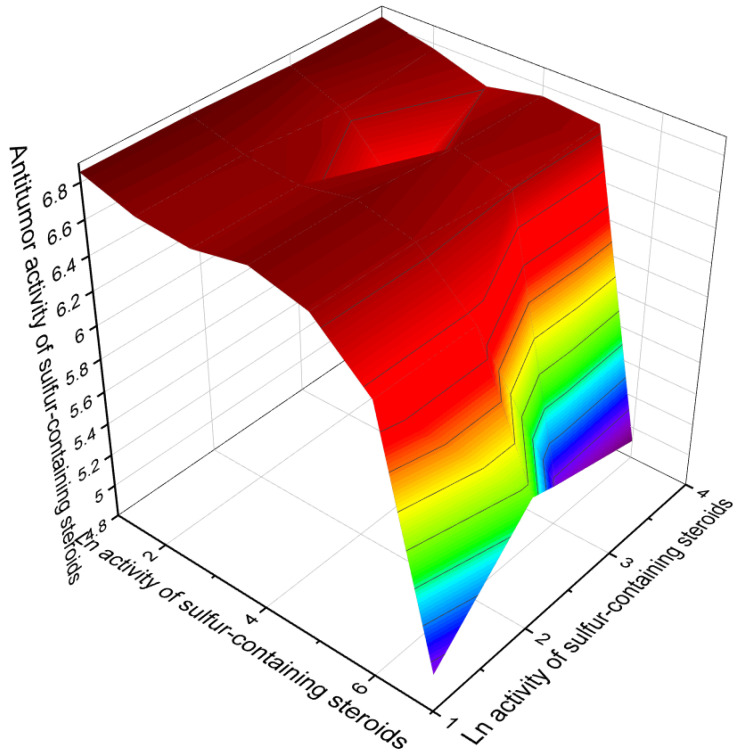
The 3D graph shows the predicted and calculated antitumor activity of selected sulfur-containing steroids (compound numbers): **98** (94.7%), **99** (94.7%), **100** (92.8%) and **105** (93.3%). These steroids show the highest degree of confidence, more than 92%.

**Figure 14:**
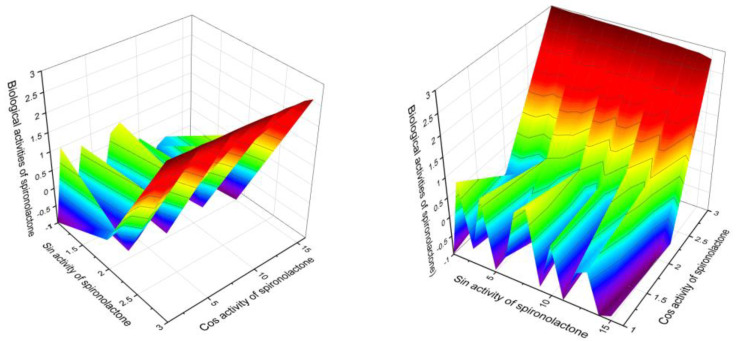
The 3D Graph (X and Y views) predicted and calculated activities of the spironolactone, which was isolated from the human urine extracts. According to PASS data, this steroid demonstrated seventeen different activities, with five activities having confidence of more than 90%. The main pharmacological properties and activities of spironolactone (**73**) are diuretic (99.1%), anti-hyperaldosteronism (96.3%), anti-hypertensive (94%), renal disease treatment (93.4%), and heart failure treatment (91.2%). As shown in numerous publications, spironolactone demonstrates properties as a potential diuretic, antihypertensive agent, or agent for the treatment of renal failure and heart failure, as well as an agent in the treatment of hyperaldosteronism (including Conn’s syndrome) and female hirsutism (due to additional antiandrogenic actions). All these properties and activities are confirmed by the PASS data as shown in this figure and shown in Table 5.

Sulfur-containing steroids are an interesting class of natural lipids, but most of these compounds exhibit weak or moderate antitumor activity. Some steroids have no antitumor activity, and only four steroids have strong antitumor activity. Figure 13 demonstrates the activity of these steroids. In addition, a sulfur-containing steroid called spironolactone (**73**) excreted from human urine is of interest. According to the PASS data, this steroid is multifunctional and demonstrates seventeen different activities. Figure 14 shows the pharmacological profile of this steroid. One feature should be noted for all sulfur-containing steroids: most of these compounds exhibit dermatological properties such as weak or moderate anti-eczematic and anti-psoriatic activity.

Most epithio steroids are classified as anabolic steroids and are used in bodybuilding diets, as well as stimulants by athletes or a specific category of people during physical or physiological stress [152,178,179,180].

**Figure 15:**
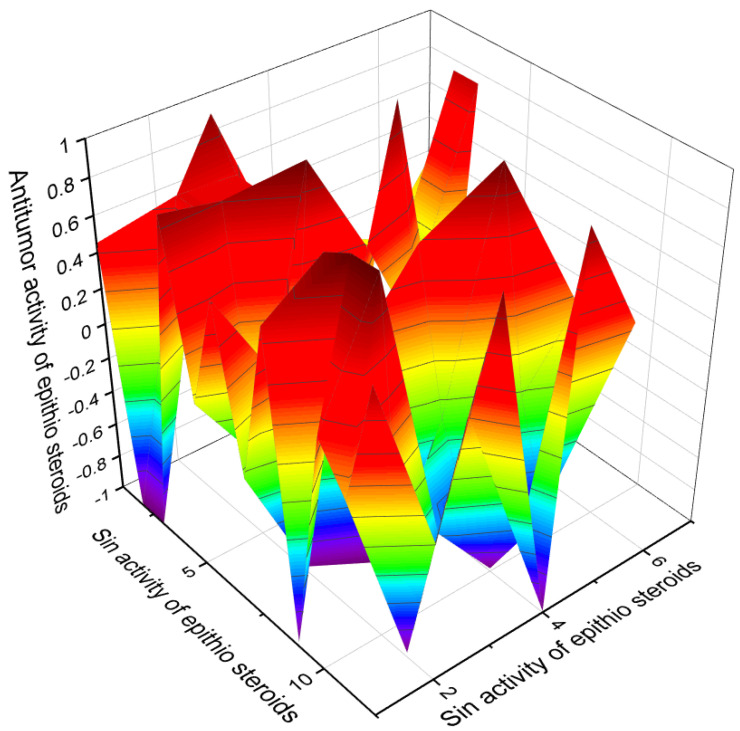
The 3D graph shows the predicted and calculated antitumor activity of selected semi- and synthetic epithio steroids (compound numbers): **122** (96.4%), **123** (96.6%), **124** (96.6%), **125** (93.2%), **126** (95.5%), **127** (96.2%), **128** (97.1%), **129** (97.0%), **131** (96.0%), **132** (93.9%), **133** (97.4%), and **138** (91.2%). These steroids show the highest degree of confidence, more than 91%.

**Figure 16:**
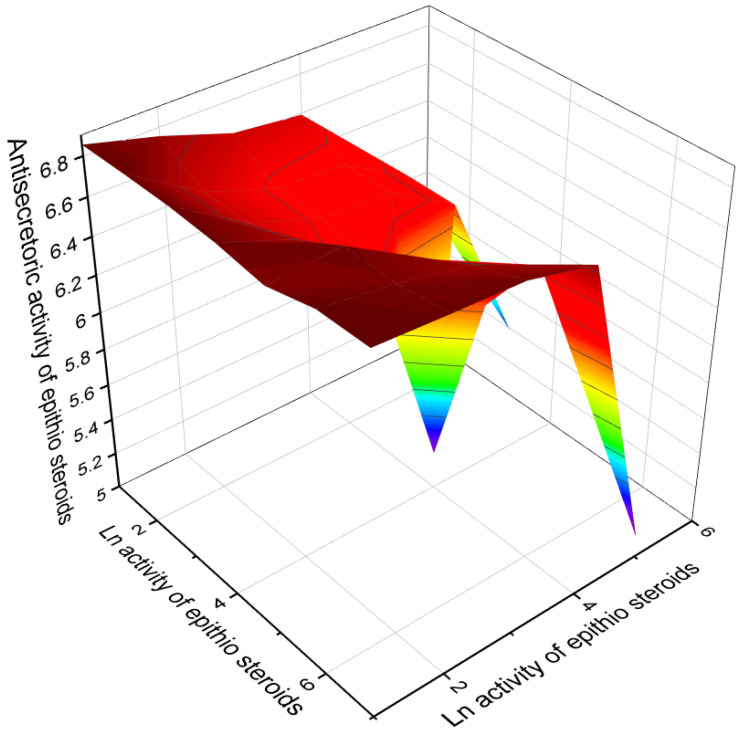
The 3D graph shows the predicted and calculated anti-secretoric activity of some an epithio steroids (compound numbers): **122** (94.8%), **123** (95.2%), **124** (95.2%), **126** (93.8%), **130** (90.6%), **131** (96.5%), and **132** (96.7%). These epithio steroids show the highest degree of confidence, more than 90%.

Several epithio steroids shown in Table 7 show strong antitumor activity, and Figure 15 shows the activity of these steroids. In addition, some epithio steroids exhibit antisecretory activity with more than 90% confidence and can be classified as antisecretory drugs. It is known that the first antisecretory drugs (antimuscarinic) were available in the 1950s, and it was not until the 1970s that the first histamine antagonist appeared. This seems to be a watershed moment in the history of acid peptic ulcer treatment. Later in the 1980s, sulfur-containing ranitidine, omeprazole, lansoprazole, rabeprazole and other proton pump inhibitors, pantoprazole, esomeprazole, and dexlanzoprazole were discovered. Their effectiveness in reducing acid secretion has been associated with side effects such as osteoporosis, malabsorption of iron and vitamin B12, hypomagnesemia, acute and chronic kidney disease, dementia, acute myocardial infarction, *Clostridium difficile* infection, and others [181,182,183]. Figure 16 demonstrates the properties of some epithio steroids as potential agents with antisecretory activity.

As shown by the PASS data, the four epithio steroids **122**, **123**, **129**, and **138** exhibit properties to increase the efficiency and improve the contraction of the heart muscle, i.e., are typical cardiotonic agents with a Pa confidence of 70% to 93.6%.

## 7. Conclusions

The review focuses on the interesting topic of sulfated steroids, which have been isolated from algae, sea sponges, soft corals, ascidians, starfish, and other marine invertebrates. In addition, sulfur-containing steroids and triterpenoids that are isolated from sedentary marine coelenterates, plants, marine sediments, crude oil, and other geological deposits are also presented in this review. Additionally, several semi-synthetic and synthetic epithiosteroids, which are a rare group of bioactive lipids that have not yet been found in nature but have a high level of antitumor activity, were included in this review for the comparative pharmacological characterization of this class of compounds.

This review presents 139 steroids and triterpenoids that exhibit a broad spectrum of biological activity. The data obtained with the PASS program show that thirteen sulfated steroids exhibit strong antitumor activity with a confidence level of more than 90%. Among the sulfur-containing steroids, only four show strong antitumor activity with a confidence level of more than 93%, although most of the presented epitosteroids show strong antitumor activity with a confidence level of 91% to 97.4%. These data are of great interest to pharmacologists and clinical physicians.

## Figures and Tables

**Table 1:** Biological activities of sulfated steroids.

No.	Antitumor & Related Activity, (Pa) *	Violation of Lipid Metabolism, (Pa )*	Additional Predicted Activity, (Pa) *
**1**	Antineoplastic (0.887) Chemopreventive (0.815)	Anti-hypercholesterolemic (0.901) Cholesterol synthesis inhibitor (0.822)	Hemostatic (0.931) Biliary tract disorders treatment (0.814)
**2**	Antineoplastic (0.906) Chemopreventive (0.816)	Anti-hypercholesterolemic (0.895) Cholesterol synthesis inhibitor (0.816)	Hemostatic (0.927) Biliary tract disorders treatment (0.819)
**3**	Antineoplastic (0.828) Chemopreventive (0.735) Apoptosis agonist (0.703)	Atherosclerosis treatment (0.786) Cholesterol synthesis inhibitor (0.733) Anti-hypercholesterolemic (0.711)	Hepatoprotectant (0.832) Anti-inflammatory (0.807) Biliary tract disorders treatment (0.717)
**4**	Antineoplastic (0.778) Chemopreventive (0.802)	Cholesterol synthesis inhibitor (0.762) Atherosclerosis treatment (0.744)	Biliary tract disorders treatment (0.752) Anti-ischemic, cerebral (0.774)
**5**	Chemopreventive (0.843) Antineoplastic (0.804)	Atherosclerosis treatment (0.721) Cholesterol synthesis inhibitor (0.744)	Biliary tract disorders treatment (0.713) Wound-healing agent (0.831)
**6**	Antineoplastic (0.880) Apoptosis agonist (0.865)	Cholesterol synthesis inhibitor (0.774) Anti-hypercholesterolemic (0.821)	Wound-healing agent (0.866) Biliary tract disorders treatment (0.764)
**7**	Antineoplastic (0.877) Chemopreventive (0.722) Apoptosis agonist (0.712)	Cholesterol synthesis inhibitor (0.776) Cholesterol synthesis inhibitor (0.755) Anti-hypercholesterolemic (0.732)	Wound-healing agent (0.852) Hemostatic (0.757) Biliary tract disorders treatment (0.741)
**8**	Chemopreventive (0.857) Antineoplastic (0.765)	Anti-hypercholesterolemic (0.884) Cholesterol synthesis inhibitor (0.766)	Biliary tract disorders treatment (0.871) Hemostatic (0.736)
**9**	Antineoplastic (0.904) Chemopreventive (0.843) Apoptosis agonist (0.721)	Atherosclerosis treatment (0.889) Cholesterol synthesis inhibitor (0.821) Anti-hypercholesterolemic (0.820)	Biliary tract disorders treatment (0.911) Anti-inflammatory (0.821) Biliary tract disorders treatment (0.724)
**10**	Chemopreventive (0.879) Apoptosis agonist (0.850) Antineoplastic (0.824)	Cholesterol synthesis inhibitor (0.862) Atherosclerosis treatment (0.862) Anti-hypercholesterolemic (0.812)	Wound-healing agent (0.872) Biliary tract disorders treatment (0.753) Hemostatic (0.731)
**11**	Chemopreventive (0.882) Antineoplastic (0.773) Apoptosis agonist (0.748)	Cholesterol synthesis inhibitor (0.869) Atherosclerosis treatment (0.858) Anti-hypercholesterolemic (0.823)	Biliary tract disorders treatment (0.762) Hemostatic (0.739)
**12**	Chemopreventive (0.873) Antineoplastic (0.778) Apoptosis agonist (0.751)	Cholesterol synthesis inhibitor (0.861) Atherosclerosis treatment (0.844) Anti-hypercholesterolemic (0.832)	Biliary tract disorders treatment (0.812) Wound-healing agent (0.775)
**13**	Antineoplastic (0.902) Chemopreventive (0.878)	Cholesterol synthesis inhibitor (0.855) Anti-hypercholesterolemic (0.831)	Wound-healing agent (0.884)

* Only activities with Pa > 0.5 are shown.

**Table 2:** Biological activities of mono sulfated steroids.

No.	Antitumor & Related Activity, (Pa) *	Violation of Lipid Metabolism, (Pa) *	Additional Predicted Activity, (Pa) *
**14**	Chemopreventive (0.828) Antineoplastic (0.798)	Anti-hypercholesterolemic (0.919) Cholesterol synthesis inhibitor (0.803)	Wound-healing agent (0.975) Biliary tract disorders treatment (0.861)
**15**	Antineoplastic (0.780) Chemopreventive (0.766)	Anti-hypercholesterolemic (0.874) Cholesterol synthesis inhibitor (0.789)	Biliary tract disorders treatment (0.955) Wound-healing agent (0.945)
**16**	Chemopreventive (0.841) Antineoplastic (0.804) Prostate disorders treatment (0.675)	Anti-hypercholesterolemic (0.907) Cholesterol synthesis inhibitor (0.818) Atherosclerosis treatment (0.724)	Wound-healing agent (0.846) Biliary tract disorders treatment (0.823) Hepatoprotectant (0.816)
**17**	Antineoplastic (0.858) Chemopreventive (0.797) Apoptosis agonist (0.719)	Anti-hypercholesterolemic (0.886) Cholesterol synthesis inhibitor (0.753) Atherosclerosis treatment (0.699)	Biliary tract disorders treatment (0.801) Wound-healing agent (0.783) Hepatoprotectant (0.771)
**18**	Antineoplastic (0.791) Prostate disorders treatment (0.692)	Anti-hypercholesterolemic (0.909); Cholesterol synthesis inhibitor (0.806);	Wound-healing agent (0.938) Biliary tract disorders treatment (0.850)
**19**	Chemopreventive (0.887) Antineoplastic (0.803)	Anti-hypercholesterolemic (0.940) Cholesterol synthesis inhibitor (0.815);	Wound-healing agent (0.952) Hepatoprotectant (0.876)
**20**	Chemopreventive (0.828) Antineoplastic (0.798)	Anti-hypercholesterolemic (0.919); Cholesterol synthesis inhibitor (0.803)	Wound-healing agent (0.975) Biliary tract disorders treatment (0.861)
**21**	Chemopreventive (0.934) Antineoplastic (0.867) Apoptosis agonist (0.857)	Anti-hypercholesterolemic (0.912) Atherosclerosis treatment (0.776) Cholesterol synthesis inhibitor (0.750)	Hemostatic (0.928) Biliary tract disorders treatment (0.764) Wound-healing agent (0.727)
**22**	Chemopreventive (0.891) Antineoplastic (0.791)	Anti-hypercholesterolemic (0.789) Cholesterol synthesis inhibitor (0.788);	Wound-healing agent (0.816) Biliary tract disorders treatment (0.807)
**23**	Antineoplastic (0.822) Chemopreventive (0.806)	Anti-hypercholesterolemic (0.926) Atherosclerosis treatment (0.804)	Biliary tract disorders treatment (0.879); Hepatoprotectant (0.764)
**24**	Antineoplastic (0.781) Chemopreventive (0.780)	Anti-hypercholesterolemic (0.929); Cholesterol synthesis inhibitor (0.762)	Biliary tract disorders treatment (0.910); Hepatoprotectant (0.876)
**25**	Chemopreventive (0.837) Antineoplastic (0.777)	Acute neurologic disorders treatment (0.808) Cholesterol synthesis inhibitor (0.719);	Wound-healing agent (0.895) Hepatoprotectant (0.888);
**26**	Chemopreventive (0.902) Apoptosis agonist (0.894) Antineoplastic (0.866);	Anti-hypercholesterolemic (0.931) Atherosclerosis treatment (0.807) Cholesterol synthesis inhibitor (0.732)	Biliary tract disorders treatment (0.844) Hepatic disorders treatment (0.818) Wound-healing agent (0.556);
**27**	Chemopreventive (0.850) Antineoplastic (0.777)	Anti-hypercholesterolemic (0.897); Cholesterol synthesis inhibitor (0.819);	Hepatoprotectant (0.879) Biliary tract disorders treatment (0.866)
**28**	Chemopreventive (0.944) Apoptosis agonist (0.808)	Cholesterol synthesis inhibitor (0.714); Atherosclerosis treatment (0.708);	Hepatoprotectant (0.872) Antifungal (0.831)
**29**	Chemopreventive (0.913) Antineoplastic (0.796) Apoptosis agonist (0.790)	Cholesterol synthesis inhibitor (0.762) Anti-hypercholesterolemic (0.726) Atherosclerosis treatment (0.717)	Wound-healing agent (0.965) Hepatoprotectant (0.933) Biliary tract disorders treatment (0.791);
**30**	Antineoplastic (0.772) Chemopreventive (0.715);	Atherosclerosis treatment (0.611) Cholesterol synthesis inhibitor (0.550)	Hepatoprotectant (0.893) Erythropoiesis stimulant (0.789);
**31**	Chemopreventive (0.882) Antineoplastic (0.803)	Anti-hypercholesterolemic (0.906) Cholesterol synthesis inhibitor (0.823)	Biliary tract disorders treatment (0.847) Hepatoprotectant (0.812);

* Only activities with Pa > 0.5 are shown.

**Table 3:** Biological activities of mono sulfated steroids.

No.	Antitumor & Related Activity, (Pa) *	Violation of Lipid Metabolism, (Pa) *	Additional Predicted Activity, (Pa) *
**32**	Antineoplastic (0.845) Chemopreventive (0.834) Apoptosis agonist (0.826);	Anti-hypercholesterolemic (0.895); Cholesterol synthesis inhibitor (0.776); Atherosclerosis treatment (0.712)	Hepatoprotectant (0.933) Biliary tract disorders treatment (0.895) Wound-healing agent (0.885)
**33**	Chemopreventive (0.821) Antineoplastic (0.805);	Cholesterol synthesis inhibitor (0.667); Atherosclerosis treatment (0.561)	Hepatoprotectant (0.859) Antiinflammatory (0.832)
**34**	Antineoplastic (0.830) Apoptosis agonist (0.792) Chemopreventive (0.783)	Atherosclerosis treatment (0.691) Cholesterol synthesis inhibitor (0.682);	Hepatoprotectant (0.968) Anti-inflammatory (0.817) Biliary tract disorders treatment (0.704);
**35**	Chemopreventive (0.777) Antineoplastic (0.779)	Anti-hypercholesterolemic (0.748) Cholesterol synthesis inhibitor (0.717)	Anti-ischemic, cerebral (0.850) Biliary tract disorders treatment (0.728)
**36**	Chemopreventive (0.821) Antineoplastic (0.765)	Cholesterol synthesis inhibitor (0.727) Anti-hypercholesterolemic (0.671)	Biliary tract disorders treatment (0.833) Wound-healing agent (0.794)
**37**	Antineoplastic (0.829)	Cholesterol synthesis inhibitor (0.618)	Anti-ischemic, cerebral (0.785)
**38**	Antineoplastic (0.813)	Atherosclerosis treatment (0.629)	Biliary tract disorders treatment (0.639)
**39**	Antineoplastic (0.823)	Atherosclerosis treatment (0.715)	Biliary tract disorders treatment (0.931)
**40**	Chemopreventive (0.924) Antineoplastic (0.837)	Anti-hypercholesterolemic (0.926) Atherosclerosis treatment (0.671)	Wound-healing agent (0.953) Hepatoprotectant (0.925)
**41**	Antineoplastic (0.854) Apoptosis agonist (0.790)	Anti-hypercholesterolemic (0.902) Atherosclerosis treatment (0.675)	Wound-healing agent (0.963) Hepatoprotectant (0.915)
**42**	Chemopreventive (0.918) Antineoplastic (0.806)	Anti-hypercholesterolemic (0.942) Cholesterol synthesis inhibitor (0.712)	Wound-healing agent (0.975) Hepatoprotectant (0.915)
**43**	Chemopreventive (0.935) Antineoplastic (0.818)	Anti-hypercholesterolemic (0.901) Atherosclerosis treatment (0.685)	Wound-healing agent (0.980) Hepatoprotectant (0.972) Biliary tract disorders treatment (0.958)
**44**	Chemopreventive (0.922) Antineoplastic (0.841)	Anti-hypercholesterolemic (0.887) Atherosclerosis treatment (0.679)	Wound-healing agent (0.977) Hepatoprotectant (0.965)
**45**	Chemopreventive (0.931) Antineoplastic (0.861);	Anti-hypercholesterolemic (0.716) Atherosclerosis treatment (0.704)	Hepatoprotectant (0.893) Wound-healing agent (0.887)
**46**	Antineoplastic (0.831)	Anti-hypercholesterolemic (0.782)	Wound-healing agent (0.888)
**47**	Chemopreventive (0.860)	Anti-hypercholesterolemic (0.850)	Wound-healing agent (0.861)

* Only activities with Pa > 0.5 are shown.

**Table 4:** Biological activities of mono-, di-, and poly-sulfated steroids.

No.	Antitumor & Related Activity, (Pa) *	Violation of Lipid Metabolism, (Pa) *	Additional Predicted Activity, (Pa) *
**48**	Chemopreventive (0.913) Antineoplastic (0.844)	Anti-hypercholesterolemic (0.825) Cholesterol synthesis inhibitor (0.678)	Hepatoprotectant (0.924) Biliary tract disorders treatment (0.727)
**49**	Antineoplastic (0.716)	Anti-hypercholesterolemic (0.865)	Biliary tract disorders treatment (0.794)
**50**	Antineoplastic (0.686)	Anti-hypercholesterolemic (0.679)	Hepatoprotectant (0.653)
**51**	Antineoplastic (0.626)	Cholesterol antagonist (0.820)	Anti-inflammatory (0.614)
**52**	Antineoplastic (0.691)	Cholesterol antagonist (0.934)	Antifungal (0.696)
**53**	Antineoplastic (0.874)	Anti-hypercholesterolemic (0.697)	Anti-inflammatory (0.718)
**54**	Antineoplastic (0.849)	Atherosclerosis treatment (0.748)	Anti-inflammatory (0.777)
**55**	Antineoplastic (0.869)	Autoimmune disorders treatment (0.762)	Angiogenesis inhibitor (0.926)
**56**	Antineoplastic (0.796)	Atherosclerosis treatment (0.646)	Anti-inflammatory (0.811)
**57**	Antineoplastic (0.836) Chemopreventive (0.712) Apoptosis agonist (0.649)	Atherosclerosis treatment (0.684) Cholesterol synthesis inhibitor (0.656) Anti-hypercholesterolemic (0.617)	Biliary tract disorders treatment (0.928) Hepatic disorders treatment (0.889) Wound-healing agent (0.888)
**58**	Antineoplastic (0.788)	Cholesterol synthesis inhibitor (0.755)	Biliary tract disorders treatment (0.843)
**59**	Antineoplastic (0.793)	Atherosclerosis treatment (0.636)	Biliary tract disorders treatment (0.713)
**60**	Antineoplastic (0.782)	Cholesterol synthesis inhibitor (0.671)	Wound-healing agent (0.933)
**61**	Antineoplastic (0.820)	Cholesterol synthesis inhibitor (0.734)	Wound-healing agent (0.942)
**62**	Antineoplastic (0.711)	Cholesterol synthesis inhibitor (0.696)	Biliary tract disorders treatment (0.963)
**63**	Antineoplastic (0.764)	Atherosclerosis treatment (0.684)	Biliary tract disorders treatment (0.957)
**64**	Chemopreventive (0.948)	Cholesterol synthesis inhibitor (0.562)	Hepatoprotectant (0.915)
**65**	Antineoplastic (0.729)	Cholesterol synthesis inhibitor (0.572)	Biliary tract disorders treatment (0.835)
**66**	Antineoplastic (0.734)	Cholesterol synthesis inhibitor (0.661)	Biliary tract disorders treatment (0.725)
**67**	Antineoplastic (0.790)	Cholesterol synthesis inhibitor (0.655)	Wound-healing agent (0.812)
**68**	Antineoplastic (0.868)	Atherosclerosis treatment (0.645)	Hepatoprotectant (0.918)
**69**	Antineoplastic (0.868)	Atherosclerosis treatment (0.645)	Hepatoprotectant (0.918)
**70**	Antineoplastic (0.748) Chemopreventive (0.692)	Cholesterol synthesis inhibitor (0.688) Atherosclerosis treatment (0.664)	Biliary tract disorders treatment (0.943) Hepatic disorders treatment (0.934)
**71**	Antineoplastic (0.767) Chemopreventive (0.704)	Cholesterol synthesis inhibitor (0.696) Atherosclerosis treatment (0.665)	Biliary tract disorders treatment (0.963) Hepatic disorders treatment (0.934);

* Only activities with Pa > 0.5 are shown.

**Table 5:** Biological activities of sulfur-containing steroids.

No.	Antitumor & Related Activity, (Pa) *	Specific Activities, (Pa) *	Additional Predicted Activity, (Pa) *
**72**	Antineoplastic (0.842)	Erythropoiesis stimulant (0.722) Prostate disorders treatment (0.688)	Anti-eczematic (0.773) Anti-seborrheic (0.733) Anti-psoriatic (0.658)
**73**	Antineoplastic (0.868)	Diuretic (0.991) Anti-hyperaldosteronism (0.963) Antihypertensive (0.940) Renal disease treatment (0.934)	Cardiotonic (0.850) Anti-ischemic, cerebral (0.822) Antiarthritic (0.741) Antithrombotic (0.691)
**74**	Antineoplastic (0.824)	Anti-osteoporotic (0.917)	Anti-seborrheic (0.845)
**75**	Antineoplastic (0.723)	Prostate disorders treatment (0.735)	Anti-eczematic (0.787)
**76**	Antineoplastic (0.736)	Prostate disorders treatment (0.745)	Anti-eczematic (0.813)
**77**	Antineoplastic (0.774)	Anesthetic general (0.774)	Anti-eczematic (0.809)
**78**	Antineoplastic (0.774)	Anesthetic general (0.774) Respiratory analeptic (0.675)	Anti-eczematic (0.809) Anti-psoriatic (0.686)
**79**	Antineoplastic (0.773) Antimetastatic (0.682)	Anti-osteoporotic (0.683)	Anti-eczematic (0.776) Anti-psoriatic (0.643)
**80**	Antineoplastic (0.729) Apoptosis agonist (0.680)	Anti-osteoporotic (0.789)	Anti-eczematic (0.782)
**81**	Antineoplastic (0.681)	Anti-osteoporotic (0.730)	Anti-eczematic (0.798)
**82**	Antineoplastic (0.664) Apoptosis agonist (0.624)	Anti-osteoporotic (0.742)	Anti-eczematic (0.771) Anti-psoriatic (0.619)
**83**	Antineoplastic (0.700)	Anti-osteoporotic (0.744)	Anti-eczematic (0.766)
**84**		Anesthetic general (0.761)	Anti-seborrheic (0.823)
**85**	Antineoplastic (0.618)	Prostate disorders treatment (0.667)	Anti-seborrheic (0.817)
**86**		Prostate disorders treatment (0.652)	Anti-seborrheic (0.810)
**87**	Antineoplastic (0.756)	Antiallergic (0.785)	Anti-psoriatic (0.818) Anti-eczematic (0.756)
**88**	Antineoplastic (0.658)	Prostate disorders treatment (0.669)	Dermatologic (0.692) Anti-eczematic (0.678)
**89**	Antineoplastic (0.730)	Anti-osteoporotic (0.696)	

* Only activities with Pa > 0.5 are shown.

**Table 6:** Biological activities of sulfur-containing steroids.

No.	Antitumor & Related Activity, (Pa) *	Specific Activities, (Pa) *	Additional Predicted Activity, (Pa) *
**90**	Antineoplastic (0.740)	Anti-osteoporotic (0.750)	Anti-eczematic (0.824)
**91**	Antineoplastic (0.773)	Hepatic disorders treatment (0.676)	Anti-seborrheic (0.692)
**92**	Antineoplastic (0.658)	Antiprotozoal (Plasmodium) (0.627)	Anti-seborrheic (0.656)
**93**		Antihypertensive (0.705)	Anti-seborrheic (0.809)
**94**		Hepatic disorders treatment (0.728)	Anti-eczematic (0.736)
**95**		Anesthetic general (0.762)	Anti-seborrheic (0.787)
**96**		Anti-osteoporotic (0.641)	Anti-eczematic (0.840)
**97**		Atherosclerosis treatment (0.680)	Anti-eczematic (0.808) Anti-psoriatic (0.649)
**98**	Apoptosis agonist (0.947) Antineoplastic (0.862)	Inflammatory bowel disease treatment (0.842)	Anti-eczematic (0.898) Anti-psoriatic (0.828) Septic shock treatment (0.619)
**99**	Apoptosis agonist (0.947) Antineoplastic (0.869)	Inflammatory bowel disease treatment (0.842)	Anti-eczematic (0.898) Anti-psoriatic (0.828) Septic shock treatment (0.619)
**100**	Apoptosis agonist (0.929) Antineoplastic (0.822)	Antipruritic (0.590)	Anti-eczematic (0.859) Anti-psoriatic (0.798)
**101**		Anti-obesity (0.750)	Anti-seborrheic (0.797) Anti-eczematic (0.696)
**102**		Anti-obesity (0.616)	Anti-seborrheic (0.782) Anti-eczematic (0.691)
**103**		Anti-obesity (0.768)	Anti-seborrheic (0.791) Anti-eczematic (0.687)
**104**		Antihypertensive (0.650)	Anti-eczematic (0.719) Anti-seborrheic (0.640)
**105**	Apoptosis agonist (0.933) Antineoplastic (0.872)	Dermatologic (0.781)	Anti-eczematic (0.847) Anti-psoriatic (0.822)
**106**	Antineoplastic (0.708)	Antihypertensive (0.655)	Anti-seborrheic (0.676)
**107**	Apoptosis agonist (0.649)	Anti-inflammatory (0.626)	Dermatologic (0.639)

* Only activities with Pa > 0.5 are shown.

**Table 7:** Biological activities of sulfur-containing steroids.

No.	Antitumor & Related Activity, (Pa) *	Specific Activities, (Pa) *	Additional Predicted Activity, (Pa) *
**108**	Antineoplastic (0.764) Antimetastatic (0.657)	Hypolipemic (0.700)	Anti-eczematic (0.766)
**109**	Antineoplastic (0.756)	Hepatic disorders treatment (0.794)	Anti-eczematic (0.753)
**110**	Antineoplastic (0.764) Antimetastatic (0.657)	Hypolipemic (0.700)	Anti-eczematic (0.766)
**111**	Antineoplastic (0.747)	Hepatic disorders treatment (0.690)	Anti-eczematic (0.739)
**112**	Antineoplastic (0.671)	Anti-osteoporotic (0.710)	Anti-eczematic (0.808) Anti-psoriatic (0.669)
**113**	Antineoplastic (0.692)	Anti-osteoporotic (0.687)	Anti-eczematic (0.803)
**114**	Antineoplastic (0.702)	Anti-osteoporotic (0.727)	Anti-eczematic (0.816)
**115**	Antineoplastic (0.766)	Hepatic disorders treatment (0.731)	Anti-seborrheic (0.836)
**116**	Antineoplastic (0.768)	Hepatic disorders treatment (0.728)	Anti-seborrheic (0.844)
**117**	Antineoplastic (0.751)	Prostate disorders treatment (0.776)	Anti-eczematic (0.792)
**118**	Antineoplastic (0.749)	Prostate disorders treatment (0.782)	Anti-eczematic (0.770)
**119**	Antineoplastic (0.834)	Anti-osteoporotic (0.717)	Anti-eczematic (0.806)
**120**	Antineoplastic (0.828)	Anti-osteoporotic (0.722)	Anti-eczematic (0.811)
**121**	Antineoplastic (0.829)	Anti-osteoporotic (0.720)	Anti-eczematic (0.809)

* Only activities with Pa > 0.5 are shown.

**Table 8:** Biological activities of epithio steroids.

No.	Antitumor & Related Activity, (Pa) *	Specific Activities, (Pa) *	Additional Predicted Activity, (Pa) *
**122**	Antineoplastic (0.964) Cytostatic (0.798) Antineoplastic (breast cancer) (0.598)	Anti-secretoric (0.948) Estrogen antagonist (0.860)	Erythropoiesis stimulant (0.760) Cardiotonic (0.729)
**123**	Antineoplastic (0.966) Cytostatic (0.681) Prostatic (benign) hyperplasia treatment (0.673)	Anti-secretoric (0.952)	Anti-inflammatory (0.754) Bone diseases treatment (0.663) Anabolic (0.648)
**124**	Antineoplastic (0.966) Cytostatic (0.681) Prostatic (benign) hyperplasia treatment (0.673)	Anti-secretoric (0.952)	Anti-inflammatory (0.754) Bone diseases treatment (0.663) Anabolic (0.648)
**125**	Antineoplastic (0.932)	Anti-secretoric (0.863) Anti-hypercholesterolemic (0.759)	Anti-eczematic (0.840) Dermatologic (0.747) Anti-psoriatic (0.659)
**126**	Antineoplastic (0.955) Cytostatic (0.676)	Anti-secretoric (0.938) Estrogen antagonist (0.807)	Anti-seborrheic (0.814) Dermatologic (0.639)
**127**	Antineoplastic (0.962)	Anti-secretoric (0.841)	
**128**	Antineoplastic (0.971) Antineoplastic (breast cancer) (0.671)	Anti-secretoric (0.861)	Anti-seborrheic (0.830) Cardiotonic (0.701)
**129**	Antineoplastic (0.970)	Estrogen antagonist (0.686) Anti-secretoric (0.677)	Cardiotonic (0.672) Dermatologic (0.649)
**130**	Antineoplastic (0.883) Cytostatic (0.661)	Anti-secretoric (0.906) Estrogen antagonist (0.750)	Anti-seborrheic (0.926) Dermatologic (0.743)
**131**	Antineoplastic (0.960) Cytostatic (0.724)	Anti-secretoric (0.965) Estrogen antagonist (0.915)	Anti-seborrheic (0.848) Anti-osteoporotic (0.729)
**132**	Antineoplastic (0.939) Cytostatic (0.787)	Anti-secretoric (0.967) Estrogen antagonist (0.946)	Anti-inflammatory (0.929) Anti-seborrheic (0.849)
**133**	Antineoplastic (0.974) Prostatic (benign) hyperplasia treatment (0.583)	Estrogen antagonist (0.870) Anti-secretoric (0.827)	Antiprotozoal (Plasmodium) (0.642) Anabolic (0.616)
**134**	Antineoplastic (0.868)	Cardiotonic (0.925) Anti-arrhythmic (0.858)	Anti-seborrheic (0.869) Anti-inflammatory (0.733)
**135**	Antineoplastic (0.780)	Anesthetic general (0.847) Anti-secretoric (0.804)	Anti-eczematic (0.811) Anti-inflammatory (0.739)
**136**	Antineoplastic (0.779) Apoptosis agonist (0.707)	Cholesterol antagonist (0.946) Anti-hypercholesterolemic (0.930)	Respiratory analeptic (0.963) Anesthetic general (0.913)
**137**	Antineoplastic (0.775)	Cholesterol antagonist (0.932) Anti-hypercholesterolemic (0.900)	Anesthetic general (0.923) Respiratory analeptic (0.919)
**138**	Antineoplastic (0.912)	Cardiotonic (0.936)	Respiratory analeptic (0.781) Anesthetic general (0.746)
**139**	Antineoplastic (0.768)	Cholesterol antagonist (0.745)	Anti-seborrheic (0.905)

* Only activities with Pa > 0.5 are shown.
